# Inhibition of *defensin A* and *cecropin A* responses to dengue virus 1 infection in *Aedes aegypti*

**DOI:** 10.7705/biomedica.5491

**Published:** 2020-08-28

**Authors:** Yda Méndez, César Pacheco, Flor Herrera

**Affiliations:** Instituto de Investigaciones Biomédicas “Dr. Francisco Triana Alonso”, Facultad de Ciencias de la Salud, Universidad de Carabobo, Maracay, Aragua, Venezuela

**Keywords:** *Aedes aegypti*, dengue virus, alpha-defensins, cecropins, *Escherichia coli*, *Aedes aegypti*, virus del dengue, alfa-defensinas, cecropinas, *Escherichia coli*

## Abstract

**Introduction:**

It is essential to determine the interactions between viruses and mosquitoes to diminish dengue viral transmission. These interactions constitute a very complex system of highly regulated pathways known as the innate immune system of the mosquito, which produces antimicrobial peptides that act as effector molecules against bacterial and fungal infections. There is less information about such effects on virus infections.

**Objective:**

To determine the expression of two antimicrobial peptide genes, *defensin A* and *cecropin A*, in *Aedes aegypti* mosquitoes infected with DENV-1.

**Materials and methods:**

We used the F_1_ generation of mosquitoes orally infected with DENV-1 and real-time PCR analysis to determine whether the *defensin A* and *cecropin A* genes played a role in controlling DENV-1 replication in *Ae. aegypti*. As a reference, we conducted similar experiments with the bacteria *Escherichia coli.*

**Results:**

Basal levels of *defensin A* and *cecropin A* mRNA were expressed in uninfected mosquitoes at different times post-blood feeding. The infected mosquitoes experienced reduced expression of these mRNA by at least eightfold when compared to uninfected control mosquitoes at all times post-infection. In contrast with the behavior of DENV-1, results showed that bacterial infection produced up-regulation of *defensin* and *cecropin* genes; however, the induction of transcripts occurred at later times (15 days). **Conclusion:** DENV-1 virus inhibited the expression of *defensin A* and *cecropin A* genes in a wild *Ae. aegypti* population from Venezuela.

## Abstract (es)

**Introducción:**

Es esencial determinar las interacciones entre los virus y los mosquitos para disminuir la transmisión viral. Estas interacciones constituyen un sistema muy complejo y muy regulado conocido como sistema inmunitario innato del mosquito, el cual produce péptidos antimicrobianos, moléculas efectoras que funcionan contra las infecciones bacterianas y fúngicas; se tiene poca información de su acción sobre los virus.

**Objetivo:**

Determinar la expresión de dos genes AMP (*defensina A* y *cecropina A)* en mosquitos *Aedes aegypti* infectados con el virus DENV-1.

**Materiales y métodos:**

Se infectaron oralmente mosquitos de generación F_1_ con DENV-1 y mediante el análisis con PCR en tiempo real se determinó el potencial papel de los genes *defensina A* y *cecropina A* en el control de la replicación del DENV-1 en *Ae. aegypti*. Como referencia, se infectaron mosquitos con *Escherichia coli*.

**Resultados:**

Los mosquitos no infectados expresaron niveles basales de los ARNm de los genes *defensina A* y *cecropina A* en diversos momentos después de la alimentación. Los mosquitos infectados experimentaron una reducción, por lo menos, de ocho veces en la expresión de estos ARNm con respecto a los mosquitos de control en todo el periodo posterior a la alimentación. En contraste con el comportamiento del virus DENV-1, los resultados mostraron que la infección bacteriana produjo una regulación positiva de los genes *defensina* y *cecropina*; sin embargo, la inducción de los transcritos ocurrió tardíamente (15 días).

**Conclusión:**

El virus DENV-1 inhibió la expresión de los genes *defensina A* y *cecropina A* en una población silvestre de *Ae. aegypti* en Venezuela.

In Venezuela, dengue is the most important arboviral disease affecting humans, and its incidence and prevalence rise annually (1). Until now, there is not a vaccine to avoid DENV infections and, therefore, vector control is the only way to restrain these disease risks (2).

Arboviruses such as DENV have to go through a series of critical steps that demand their interplay with different tissues, which lasts for days or weeks until transmission can occur (2-4). These tissues represent barriers that restrict virus growth through, among others, immune molecules with antipathogenic activity that belong to a very complex system of highly regulated pathways called the innate immune system of mosquitos. Toll, IMD, JAK/STAT, and RNAi are the primary immune signaling pathways (2-5).

Different strategies to diminish viral transmission have been considered, among them, the use of genetically engineered vectors and natural symbionts like *Wolbachia* (6,7). Any strategy to control dengue transmission should consider the interactions between viruses and mosquitoes, especially, their innate immune system.

Toll and IMD pathways produce effector molecules such as the antimicrobial peptides, low molecular-weight proteins well known for their action against bacterial and fungal infections, although there is less information regarding their effect on viral infections. Reports suggest that dengue virus infection is controlled by the toll pathway in mosquitoes (8) and that together with the IMD pathways they upregulate the Sindbis (9) and the DENV-2 viruses in mosquitoes (10,11). However, other studies have evidenced the inhibition of toll's innate immune response in salivary glands infected by DENV-2 with 3’UTR substitutions associated with high epidemiological fitness and enhanced production of infectious saliva (12).

In our study, we found that the expression of *defensin A* and *cecropin A* genes, two antimicrobial peptide genes mediated by the toll pathway, was significantly reduced in *Ae. aegypti* mosquitoes infected with DENV-1 suggesting that the infection progresses by suppressing the toll pathway.

## Materials and methods

### Mosquito collection

We collected *Ae. aegypti* mosquitoes as larvae from Maracay, Venezuela, and then obtained their F_1_ generation.

### Dengue virus, bacteria, and infection processes

We used a DENV-1 isolate (LAR23644) recovered from a patient in Maracay in 2007 for the infection assays. Viruses were serially passaged in *Ae. albopictus* C6/36 cells, the infected supernatants were then harvested, titered via plaque-forming assay, and frozen at -80 °C. The viral titer was 4,8 x 10^5^ PFU/ml. For oral infection experiments, we mixed viral stocks 1:1 with human red blood cells washed with PBS and fed to mosquitoes (sugar starved for 24 h) via membrane feeders. Some groups of mosquitoes were fed only on human red blood cells. Immediately post-feeding, fully engorged specimens were transferred to new cages held under standard rearing conditions and provided with sucrose.

At different times after feeding, ≈30 mosquitoes were collected each time. At early times (5 and 24 hours) we checked if the virus was inactivated by some antiviral defense mechanism present in the mosquitoes’ guts while at later times (10 and 15 days), we aimed at detecting viral replication in their bodies.

To determine the percentage of virus infection, dissemination, and potential transmission in the vector, 50 individual mosquitoes’ abdomen (fed with the virus similarly as before and collected 15 days later) were dissected to check for infection, their legs and wings for dissemination, and their salivary glands for potential transmission. It is known that the only way to measure transmission is by analyzing the saliva of the mosquitoes, for which the viruses in these last tissues are potentially transmittable (13). Prior to their lysis, all the tissues were washed three times with 200 μl of PBS to discard any contamination. Mosquitoes were stored at -80 °C.

Similar infections were carried out with *E. coli* cultured in OD_600_ 0.8, pelleted, washed, and resuspended in PBS. The bacteria culture was mixed with human red blood cells in equal proportions, and then we applied the methodological procedure used for viral infection.

### Detection and typing of dengue viruses in Aedes aegypti

RNA extraction, detection, and typing of dengue viruses in pools of whole bodies or in dissected samples of *Ae. aegypti* were performed according to Urdaneta, *et al.* (14).

### *Quantitative RT*-*PCR* (qPCR) *for measuring gene expression*

Gene expression was determined by relative quantification relating the qPCR signal of the *defensin A* or *cecropin A* gene transcript in a mosquito group fed on virus or bacteria mixed with human red blood cells and that of a control group (calibrator) fed only on human red blood cells. qPCR was conducted in a reaction volume of 25 µl in a 96-well plate containing 0.5 µg of template based on the initial RNA concentration and 200 nM forward and reverse primers using real-time Go *Taq* qPCR™ (Promega Corporation, USA) on a 7500 Real-Time PCR System™ (Applied Biosystems, Massachusetts, USA) using the following program: 2 minutes of preincubation at 95 ºC followed by 40 30-s cycles at 95 ºC and one minute at 60 ºC. The designed specific primers used were: *Defensin A* gene (sense: 5’-AACTGCCGGAGGAAACCTAT-3’; antisense: 5’-TCTTGGAGTTGCAGTAACCT-3’) and *cecropin A* gene (sense: 5'-CGAAGTTATTTCTCCTGATCG-3'; antisense: 5'-AGCTACAACAGGAAGAGCC-3'). To normalize the data, we used the *α-tubulin* gene (sense: 5’-GCGTGAATGTATCTCCGTGC-3’; antisense: 5'-AGCTACAACAGGAAGAGCC-3') s an endogenous reference.

We assessed *α-tubulin, defensin A*, and *cecropin A* primer pairs and we found the following for each: The observed efficiency was near to 100% (figure 1S), the amplification specificity was displayed through the production of a unique peak in the melt-curve analysis (figure 2S), which was corroborated by sequencing the PCR products from each gene in both directions using the PCR primers (data not shown). The sequencing reactions were performed with the ABI PRISM BigDye Terminator™, version 3.1 Cycle Sequencing Kit on an Applied Biosystems genetic analyzer, Model ABI 3130XL. Therefore, the 2^-ΔΔCt^ method of relative quantification was used to appraise relative gene expression.

**Figure 1S:**
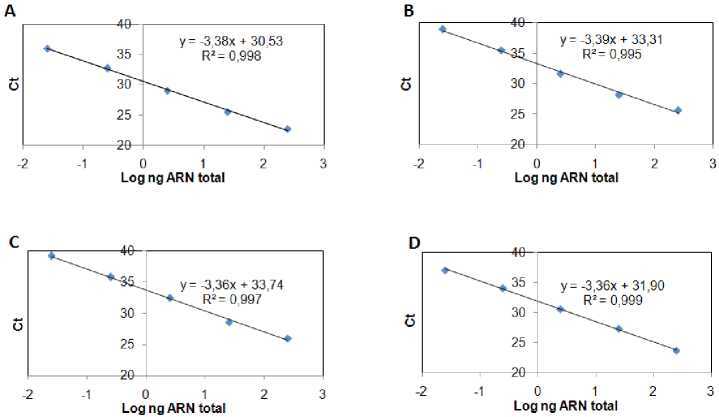
Efficiency of gene amplification. qPCR amplification efficiencies of α-tubulin, defensin A, and cecropin A genes. Each gene DNA was diluted in serial 10-fold ranges and the cycle threshold (Ct) value at each dilution was measured. The Ct represents the average of two independent experiments done by triplicate. Then, a curve was obtained for: a) α-tubulin, b) defensin A or c) cecropin A gene from which qPCR efficiencies (E) were assessed. The slopes of the curves were used to calculate E according to the equation: E = 10(-1/slope) - 1 × 100, where E = 100 corresponds to a 100% efficiency. The amplification efficiencies obtained for the genes after applying the equation were: A) *α-tubulin* gene, 97.3% B) *defensin A* gene, 97.2% C) *cecropin A* gene, 98.5%

**Figure 2S:**
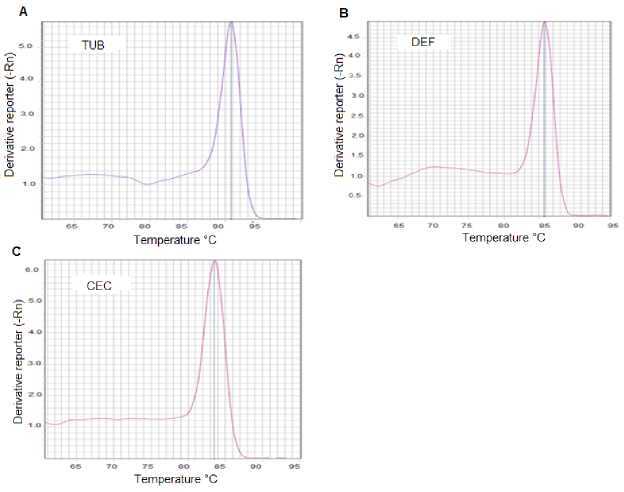
DNA qPCR melting curve analysis for the detection of gene specificity. Three fragments, the first from the α-tubulin gene, the second from the defensin A, and the last from the cecropin A gene were synthesized by qPCR using specific primers for each gene. The resulting products were subjected to post-PCR melt analysis. Only one peak was detected with primers for A) α-tubulin gene; B) defensin A gene or C) cecropin A gene.

We used the control and virus-infected pool samples (≈30 mosquitoes/pool) at different times after feeding (5 h, 24 h, 10 days, and 15 days) in the qPCR reaction (a total of 8 pools: ≈240 mosquitoes). The control values were very close at all times, so we took their average as the calibrator. Each qPCR experiment was repeated three times with three replicates of each one. Similar experiments were carried out with *E. coli.* The average and standard deviation (SD) of the C_T_s from the three replicates were determined and the average was only approved if the SD was <0.38 (15). Repeatability and reproducibility were calculated by a percent coefficient of variance (% CV) within and between assays respectively (table 3S).

**Table 3S:** Repeatability of qPCR assay for *defensin A* gene in Escherichia coli-infected *Aedes aegypti* mosquitoes

**Sample**	**Replicated runs (Ct)**			**Mean**	**SD**	**Repeatability (CV)**	**%** **CV**
	**1**	**2**	**3**				
Calibrator	27	27.2	26.8	27.0	0.2	0.01	0.7
5 h	25.4	25.7	25.4	25.5	0.2	0.01	0.7
24 h	27.9	27.1	27.5	27.5	0.4	0.01	1.5
10 d	25.9	26.9	26.6	26.5	0.5	0.02	1.9
15 d	22.8	23.1	22.8	22.9	0.2	0.01	0.8

SD: standard deviation; CV: coefficient of variance

We calculated N-fold copy numbers of the *Ae. aegypti defensin A* and *cecropin A* gene transcripts relative to the control in each assay using geometric means for the three experiments.

## Results

### Stability and replication of DENV-1

We determined whether the DENV-1 was stable at early post-infection (dpi) times (5 and 24 hours) and replicated at later ones (10 and 15 days) in the mosquitoes using RT-PCR amplification followed by agarose gel electrophoresis analysis of the products. Figure 1 shows the presence of DENV-1 with the cDNA band at the 482 bp position at all time points under study. The replication was further corroborated in the dissected samples of 50 individual mosquitoes with 70% and 100% viral infection and a dissemination efficiency 15 days post-infection. Regarding the virus present in the salivary glands, it also replicated (45%) and evidenced potential transmission efficiency (table 9S).

**Figure 1:**
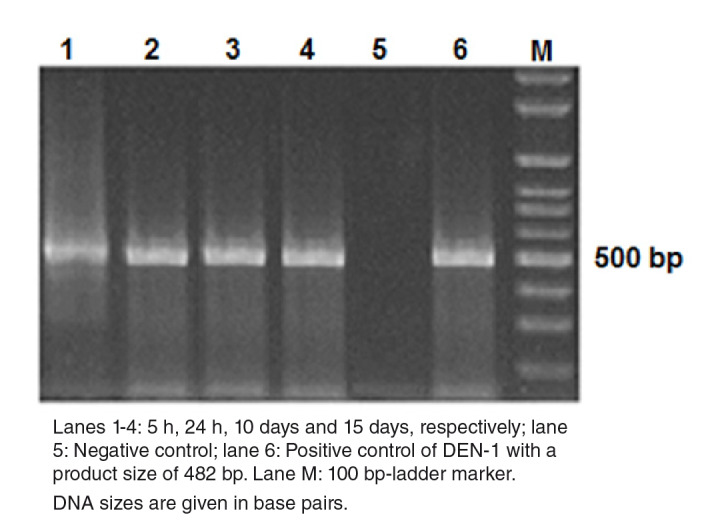
Detection of DEN-1 in *Aedes aegypti* by gel electrophoresis on a 2% agarose gel. DNA amplicons generated by RT-PCR of the RNA extracted from dengue viruses in *Aedes aegypti* at different times post infection. Lanes 1-4: 5 h, 24 h, 10 days and 15 days, respectively; lane 5: Negative control; lane 6: Positive control of DEN-1 with a product size of 482 bp. Lane M: 100 bp-ladder marker. DNA sizes are given in base pairs.

**Table 9S:** Rates of infection, dissemination and potential transmission of the DENV-1 in Ae. aegypti mosquitoes

**Infection (%)^a^ Dissemination (%)^b^ P. transmission (%)^c^**
70 (35/50) 100 (35/35) 45.7 (16/35) (p<0,01)*
^a^ Rate of infection: Number with DENV virus-positive abdomens/number tested
^b^ Rate of dissemination: Number with DENV virus-positive legs and wings/number with DEN
virus-positive abdomens
^c^ Rate of potential transmission: Number with DENV virus-positive heads/number with DEN
virus-positive abdomens
* Open Epi Versión 3.03 (Dean AG, Sullivan KM, Soe MM. OpenEpi: Open Source
Epidemiologic Statistics for Public Health, Version. www.OpenEpi.com, updated 2013/04/06,
accessed 2020/04/03) was used for statistical analysis

### Inhibition of defensin and cecropin mRNA by DENV-1

The relative expression levels of *defensin A* and *cecropin A* genes in DENV-1*-*infected *Ae. aegypti* mosquitoes as compared to the calibrator are shown in figure 2A with both mRNA detectable in control mosquitoes; however, a significant decrease in abundance occurred at all time points measured with at least five to eight-fold fewer amounts of *defensin* and *cecropin* mRNA, respectively, in mosquitoes infected with DENV-1.

**Figure 2:**
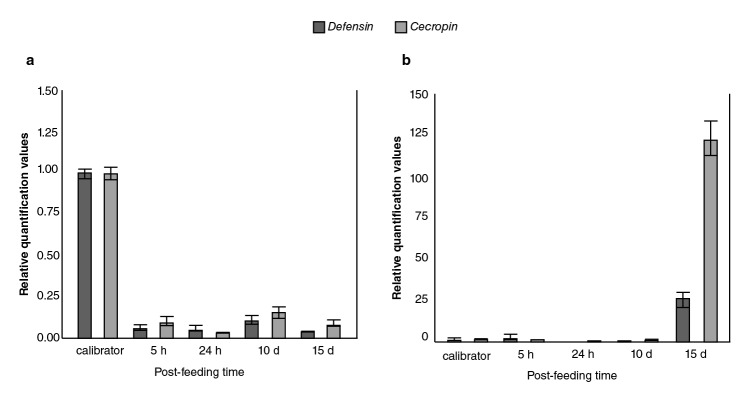
Comparison of immune responses to DENV-1 and *Escherichia coli* bacteria in field-collected *Aedes aegypti.* Averaged data from three independent real-time qPCR experiments were used to assess the expression of each of the selected immune genes in the *Aedes aegypti* mosquitoes infected with the DEN-1 virus (a) or *Escherichia coli* bacteria (b) with the host *α-tubulin* as an internal reference control to normalize the data. For each pathogen, the control values for both genes at all the time points were very similar. In every case, the average of all these values was used as the calibrator. The 2-^ΔΔCT^ method was used to calculate fold change for each gene.

### Induction of defensin and cecropin mRNA by bacteria

The response of the field population of *Ae. aegypti* mosquitoes to bacteria was contrary to the previously described viral response given that, as expected, the bacterial infection did not produce down-regulation in any of the genes (figure 2B); however, the induction of transcripts occurred at later times (15 days).

## Discussion

There is a discrepancy regarding the reaction of mosquitoes’ immune system in the presence of DENV-1. The response may be stimulation (2,8,10,11) or suppression *in vivo* (9,16-18) and *in vitro* (19,20). Such discrepancy probably depends on the viral strain used, the genetic history of the vector, and the mode of transmission (3,21). We found a reduced expression of *defensin A* and *cecropin A* genes using the F_1_ generation of wild mosquitoes infected with DENV-1. Similar results were reported with DENV-2-infected field *Ae. aegypti* populations (6).

The specific molecular mechanism by which DENV acts remains uncharacterized. The virus may be able to knock down the expression of some factor needed to induce the expression of *defensin* and *cecropin* mRNA, similar to the role reported for the *Ae*FaDD protein in *Ae. aegypti* (22). Alternatively, the DENV may directly target and inhibit the transcription of both genes.

The suppression of the innate immune responses of mosquitoes found in this study was time-independent contrary to other reports using similar times: 1, 2, 7, and 14 days (17), which implies that DENV may exert continuous immunomodulatory activity in mosquitoes, or for some period of time. This is critical for defining the vector competence of local mosquitoes, as well as dengue transmission intensity in a particular area.

As expected, the bacterial infection did not produce down-regulation of the defensin and cecropin genes (23,24); however, the induction of the transcripts occurred at later times (figure 2B). These data could indicate that the capacity of these wild *Ae. aegypti* mosquitoes to mount a highly effective production of defensin and cecropin to control invading bacteria would take the time probably required to inactivate bacterial growth factors.

In conclusion, DENV-1 inhibits the expression of *defensin A* and *cecropin A* genes in a wild *Ae. aegypti* population from Maracay city in Venezuela. The way the virus participates in this inhibitory mechanism and the viral effector molecules acting in it are still to be determined.

## Acknowledgments

To Nancy Moreno for her critical reading of the manuscript; to Karem Flores and to the *Dirección de Malariología y Saneamiento Ambiental* staff in Maracay, Estado Aragua, Venezuela, for the collection and rearing of mosquitoes; to Daria Camacho for providing the virus strain, and to. Irene Bosch for assistance with the English language editing of this manuscript.

## Supplementary files

**Figure 3S:**
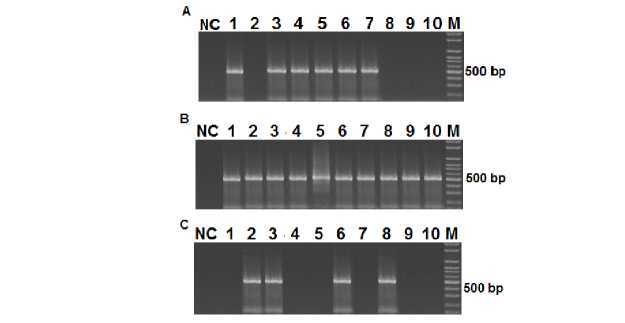
Infection, Dissemination and Potential Transmission of DENV-1 in Aedes aegypti mosquitoes. Mosquitoes (50) were infected with DENV-1 (1.6 x 105 PFU/ml) and after 15 days, RNA was extracted from different tissues of individual mosquitoes and visualized using nested PCR followed by electrophoresis on a 2% agarose gel and SYBR Green staining. The 482 bp band corresponds to the DENV-1 amplification product from abdomens (A), wings and legs (B), and heads (C).

(Figure 1S), (Figure 2S), (Figure 3S), (Table 1S), (Table 2S), (Table 3S), (Table 4S), (Table 5S), (Table 6S), (Table 7S), (Table 8S), (Table 9S)

**Table 1S:** Repeatability of qPCR assay for *defensin A* gene in DEN 1-infected *Aedes aegypti* mosquitoes

**Sample**	**Replicated runs (Ct)**			**Mean**	**SD**	**Repeatability**	**%** **CV**
	**1**	**2**	**3**				
Calibrator	25.6	26.8	27.1	26.5	0.8	0.03	3.0
5 h	31.5	32.4	31.6	31.8	0.5	0.02	1.5
24 h	30.5	31.2	30.6	30.8	0.4	0.01	1.2
10 d	31.8	31.5	31.8	31.7	0.2	0.01	0.5
15 d	32.6	31.3	32.6	32.2	0.8	0.02	2.3

SD: standard deviation; CV: coefficient of variance

**Table 2S:** Repeatability of qPCR assay for *cecropin A* gene in DEN 1-infected *Aedes aegypti* mosquitoes

**Sample**	**Replicated runs (Ct)**			**Mean**	**SD**	**Repeatability (CV)**	**%** **CV**
	**1**	**2**	**3**				
Calibrator	23	24	22	23.0	1.0	0.04	4.3
5 h	26.9	27.5	27.4	27.3	0.3	0.01	1.2
24 h	29.2	29.5	29.2	29.3	0.2	0.01	0.6
10 d	26.9	26.7	26.4	26.7	0.3	0.01	0.9
15 d	28.7	28.8	28.8	28.8	0.1	0.00	0.2

**Table 4S:** Repeatability of qPCR assay for *cecropin A* gene in Escherichia coli-infected *Aedes aegypti* mosquitoes

**Sample**	**Replicated runs (Ct)**			**Mean**	**SD**	**Repeatability (CV)**	**%** **CV**
	**1**	**2**	**3**				
Calibrator	26	26	25	25.7	0.6	0.02	2.2
5 h	24	25	24	24.3	0.6	0.02	2.4
24 h	25.1	25.9	25.8	25.6	0.4	0.02	1.7
10 d	24.8	25.3	25.3	25.1	0.3	0.01	1.1
15 d	23.9	24	24.4	24.2	0.4	0.01	1.5

SD: standard deviation; CV: coefficient of variance

For all tables, the repeatability was calculated as the percent coefficient of variance (%CV) of Ct triplicate values of a sample within a single experiment.

**Table 5S:** Reproducibility of qPCR assay for *defensin A* gene in DEN 1-infected *Aedes aegypti* mosquitoes

**Sample**	**Run (% CV)**			**Mean**	**SD**	* Reproducibility run 1 ~ run 2 ~ run 3 (% CV)
	**1**	**2**	**3**			
Calibrator	3.0	2.8	2.9	2.9	0.10	0.034
5 h	1.5	1.4	1.5	1.5	0.08	0.051
24 h	1.2	1.4	1.2	1.3	0.11	0.084
10 d	0.5	0.4	0.5	0.5	0.08	0.155
15 d	2.3	2.2	2.3	2.3	0.07	0.030

CV: coefficient of variance; SD: Standard deviation

For all tables, the reproducibility was calculated as the percent coefficient of variance (%CV) of Ct triplicate values of a sample between assays.

**Table 6S:** Reproducibility of qPCR assay for *cecropin A* gene in DEN 1-infected *Ae. aegypti* mosquitoes

**Sample**	**Run (% CV)**			**Mean**	**SD**	* Reproducibility run 1 ~
	**1**	**2**	**3**			**run 2 ~ run 3 (% CV)**
Calibrator	0.7	0.9	0.7	0.8	0.11	0.135
5 h	0.7	0.8	0.7	0.7	0.06	0.089
24 h	1.5	1.4	1.6	1.5	0.10	0.070
10 d	1.9	1.9	1.8	1.9	0.07	0.038
15 d	0.8	0.9	0.8	0.8	0.07	0.090

SD: standard deviation; CV: coefficient of variance; CV: coefficient of variance

**Table 7S:** Reproducibility of qPCR assay for *defensin A* gene in Escherichia coli-infected *Ae. aegypti* mosquitoes

**Sample**	**Run (% CV)**			**Mean**	**SD**	* Reproducibility run 1 ~
	**1**	**2**	**3**			**run 2 ~ run 3 (% CV)**
Calibrator	2.2	2.1	2.2	2.2	0.08	0.035
5 h	2.4	2.6	2.4	2.5	0.12	0.051
24 h	1.7	1.7	1.6	1.7	0.06	0.035
10 d	1.1	1.2	1.2	1.2	0.03	0.025
15 d	1.5	1.3	1.5	1.4	0.11	0.075

CV: coefficient of variance; SD: Standard deviation

**Table 8S:** Reproducibility of qPCR assay for cecropin A gene in Escherichia coli-infected Aedes aegypti mosquitoes

**Sample**	**Run (% CV)**			**Mean**	**SD**	* Reproducibility run 1 ~
	**1**	**2**	**3**			**run 2 ~ run 3 (% CV)**
Calibrator	4.3	4.3	4.2	4.3	0.08	0.018
5 h	1.2	1.3	1.3	1.3	0.07	0.055
24 h	0.6	0.5	0.6	0.6	0.06	0.098
10 d	0.9	0.8	0.8	0.8	0.08	0.098
15 d	0.2	0.2	0.1	0.2	0.06	0.347

CV: coefficient of variance; SD: Standard deviation

### Repeatability

### Reproducibility
